# Understanding Hospital Readmissions: Insights, Patterns, and Interventions for Improvement in Chronic Kidney Disease

**DOI:** 10.7759/cureus.59524

**Published:** 2024-05-02

**Authors:** Arun Surasura, Bobbadi Gajendra Siva Krishna Pavan Kumar, Sravani Chinamanagonda, Divya Durga, Sahithi Gubbala

**Affiliations:** 1 Internal Medicine, NRI Medical College and General Hospital, Guntur, IND; 2 Internal Medicine, NRI Academy of Medical Sciences, Guntur, IND; 3 Medicine, NRI Medical College and General Hospital, Guntur, IND

**Keywords:** hospital readmissions, interventions, comorbidities, demographics, chronic kidney disease

## Abstract

Background: Hospital readmissions among chronic kidney disease (CKD) patients pose substantial challenges in healthcare, impacting both patients and healthcare systems. Understanding the patterns and determinants of CKD-related readmissions is crucial for devising effective interventions.

Objective: This research aimed to investigate the factors contributing to hospital readmissions among CKD patients, identify the primary reasons for readmissions, and explore potential interventions to mitigate readmission risks.

Methods: A retrospective analysis was conducted among a cohort of 300 CKD patients over an 18-month period at a tertiary care unit specializing in nephrology services. Data on demographics, CKD stages, comorbidities, reasons for readmissions, and lengths of hospital stays were analyzed. Logistic regression models were employed to identify predictors of readmissions.

Results: Advancing CKD stages were associated with increased readmission rates, with higher rates observed in older patients. Cardiovascular complications and acute kidney injury were prominent reasons for readmissions. Age, comorbid conditions, and previous hospitalizations emerged as significant predictors of readmissions. Lengths of hospital stays during readmissions were also correlated with CKD stages.

Conclusion: The research underscores the complex interplay of demographic and clinical factors contributing to hospital readmissions among CKD patients. Tailored interventions addressing disease severity, comorbidities, and patient-specific characteristics are pivotal in reducing readmission risks and enhancing care outcomes for this population.

## Introduction

Chronic kidney disease (CKD) stands as a prevalent and burdensome condition globally, affecting millions and posing substantial challenges to healthcare systems [1]. Among its manifold complications, hospital readmissions represent a critical concern, exerting a profound impact on both patients and healthcare systems. Readmissions not only heighten healthcare costs but also signify a failure in the continuum of care, emphasizing the need for a more comprehensive understanding and effective management of CKD-related readmissions [2]. This paper aims to delve into the intricate landscape of hospital readmissions within the context of CKD, scrutinizing the patterns, determinants, and potential interventions crucial for ameliorating the burden of recurrent hospitalizations among CKD patients. The multifaceted nature of CKD, often accompanied by comorbidities such as hypertension, diabetes mellitus, and cardiovascular diseases, complicates disease management and significantly contributes to the recurrence of hospital admissions [3]. The interplay of these comorbidities necessitates a deeper exploration of their influence on readmission rates among CKD patients.

Moreover, socio-economic factors intertwined with healthcare accessibility and disparities in healthcare provision play a pivotal role in shaping readmission patterns [4]. Variations in readmission rates among different demographic groups highlight the need for tailored interventions to address disparities and improve outcomes for vulnerable populations [5]. The elderly, for instance, often exhibit higher readmission rates due to complex medical needs and potential social challenges, necessitating a targeted approach to their care [6]. Epidemiological data underscore the magnitude of the problem, revealing a substantial prevalence of CKD-related hospital readmissions. The frequent readmissions, often stemming from exacerbations of CKD, complications from comorbid conditions, or inadequate management post-discharge, emphasize the exigency of effective interventions to curtail this cycle [7].

In parallel, healthcare systems worldwide are grappling with the escalating costs associated with CKD-related hospital readmissions. This necessitates a reevaluation of care delivery systems, emphasizing transitional care programs, patient education initiatives, and enhanced interdisciplinary collaboration to bridge the gaps in care and reduce readmission rates [8]. Transitional care programs have shown promise in ensuring a smoother transition from hospital to home, thereby mitigating readmission risks [9]. Equipping patients with self-management skills and facilitating better communication between healthcare providers and patients prove pivotal in averting avoidable readmissions [10]. As such, this paper endeavors to synthesize existing literature, analyze retrospective data, and synthesize evidence-based interventions to delineate a comprehensive understanding of CKD-related hospital readmissions. By scrutinizing the intricate interplay of medical, socio-economic, and healthcare delivery factors, this research aims to pave the way for targeted strategies to minimize readmission rates among CKD patients.

## Materials and methods

The research was conducted at a tertiary care unit specializing in nephrology services over an 18-month period. The study aimed to analyze hospital readmission patterns among a cohort of CKD patients and identify associated factors contributing to recurrent hospitalizations.

The research cohort comprised 300 patients, including those diagnosed with CKD stages 3-5, aged 18 years or older, who received treatment and follow-up care at the tertiary care unit during the research period. Patients with acute kidney injury, end-stage renal disease on dialysis, or those undergoing kidney transplantation were excluded from the research to maintain homogeneity within the CKD population under investigation. Retrospective data analysis was conducted, leveraging electronic health records (EHRs), hospital admission, and discharge databases. Patient demographics, including age, gender, socio-economic status, and relevant clinical variables such as CKD stage, comorbid conditions (hypertension, diabetes mellitus, cardiovascular diseases), and previous hospitalizations, were extracted from the EHRs.

The primary outcome assessed was the incidence and reasons for hospital readmissions among CKD patients within 30 days of discharge during the research period. Additionally, the secondary outcomes included the length of hospital stay during readmissions and the association between readmissions and specific patient characteristics. Statistical analysis was performed using appropriate methodologies, such as logistic regression models, to identify predictors of readmissions, adjusting for potential confounding variables. Subgroup analyses were conducted based on CKD stages to assess variations in readmission rates and associated factors across different disease severities.

Ethical approval was obtained from the NRI Medical College and General Hospital with the institutional review board (IRB) number IEC/005/2024 prior to data collection and analysis, ensuring compliance with ethical guidelines and patient confidentiality. All data were anonymized to safeguard patient privacy and confidentiality.

The chi-squared test was utilized to analyze categorical variables, such as socio-economic status and previous hospitalizations, and their association with hospital readmissions. P-values of <.05 were considered to be statistically significant. Analyses were performed using SAS version 9.4 (SAS Institute, Cary, North Carolina) and R 3.1.1 (R Foundation for Statistical Computing, Vienna, Austria).

## Results

The analysis of demographic characteristics among different CKD stages revealed notable patterns. The mean age varied significantly across CKD stages, with patients in higher stages being older. For instance, patients in CKD Stage 5 had a notably higher mean age of 68.1 years compared to Stage 3 (55.6 years) and Stage 4 (62.4 years) (P<0.001). Furthermore, a disparity in gender distribution was observed, with earlier CKD stages having a higher proportion of male patients, whereas Stage 5 CKD had a higher representation of female patients (P=0.023). The readmission rate increased significantly with advancing CKD stages, indicating a correlation between disease severity and the likelihood of readmission (P<0.001) (Table 1).

**Table 1:** Demographic characteristics and readmission rates among CKD patients A chi-squared test (χ² test) was used, with a statistical significance of <0.05.

Demographic Variables	CKD Stage 3	CKD Stage 4	CKD Stage 5	P-value
Age (years), mean (SD)	55.6 (8.3)	62.4 (7.9)	68.1 (6.5)	<0.001
Gender (male/female)	60/40	45/55	30/70	0.023
Socio-economic status	Low	Moderate	High	0.117
Readmission rate (%)	25	32	42	<0.001

The primary reasons for readmissions among CKD patients within 30 days post-discharge were explored, with cardiovascular complications and acute kidney injury emerging as the most prevalent causes. Cardiovascular complications accounted for 25.4% of readmissions, whereas acute kidney injury contributed to 18.5% of readmissions, underscoring the critical nature of these conditions in driving hospital readmissions among CKD patients (Table 2).

**Table 2:** Primary reasons for hospital readmissions among CKD patients

Reasons for Readmissions	Frequency (n)	Percentage (%)
Acute kidney injury	35	18.5
Cardiovascular complications	48	25.4
Infection	27	14.3
Medication-related issues	22	11.6
Others	68	30.2

Logistic regression analysis unveiled significant predictors of hospital readmissions among CKD patients. For every 10-year increase in age, there was a 25% higher odds of readmission (P=0.003). The presence of comorbidities exhibited more than double the odds of readmission (P<0.001), emphasizing the influence of concurrent health conditions on readmission risks. Moreover, a history of previous hospitalizations nearly doubled the odds of readmission (P<0.001), underscoring the impact of prior healthcare utilization on recurrent admissions (Table 3).

**Table 3:** Predictors of hospital readmissions among CKD patients A chi-squared test (χ² test) was used, with a statistical significance of <0.05. CI: confidence interval.

Predictors	Odds Ratio (95% CI)	P-value
Age (per 10 years)	1.25 (1.08-1.45)	0.003
Comorbidity (yes/no)	2.14 (1.72-2.67)	<0.001
Socio-economic status	1.05 (0.92-1.20)	0.364
Previous hospitalizations	1.98 (1.61-2.43)	<0.001

The analysis also assessed the duration of hospital stays during readmissions across CKD stages. The length of stay increased as CKD stages progressed, with Stage 5 CKD patients experiencing significantly longer hospital stays (8.5 days) compared to Stage 3 (4.7 days) and Stage 4 (6.2 days). This suggests a correlation between disease severity and the duration of hospitalization during readmissions among CKD patients (Table 4).

**Table 4:** Length of hospital stay during readmissions among CKD patients

CKD Stage	Mean Length of Stay (Days)	Standard Deviation	P-value
Stage 3	4.7	1.2	0.001
Stage 4	6.2	1.5	
Stage 5	8.5	2	

In summary, the research elucidated the intricate relationship between demographic factors, reasons for readmissions, predictors of readmission, and lengths of hospital stays among CKD patients. These findings underscore the importance of tailored interventions addressing age-related risks, comorbid conditions, and disease severity to mitigate hospital readmissions and improve care outcomes for CKD populations.

## Discussion

The findings from this research shed light on several crucial aspects concerning hospital readmissions among CKD patients, corroborating and extending upon existing literature. The observed correlation between advancing CKD stages and increased readmission rates aligns with previous research highlighting the progressive nature of CKD and its impact on healthcare utilization [1]. Additionally, the association between older age and higher readmission rates among CKD patients resonates with studies emphasizing age as a significant predictor of adverse outcomes in this population [2].

The predominant causes of readmissions, particularly cardiovascular complications and acute kidney injury, echo the multifaceted nature of CKD, where both renal and cardiovascular comorbidities contribute significantly to adverse outcomes and hospital admissions [3]. This emphasizes the need for holistic management strategies addressing both renal and cardiovascular health to reduce readmission risks among CKD patients. The identified predictors of readmissions, including comorbidities and previous hospitalizations, corroborate existing evidence, highlighting the influence of concurrent health conditions and healthcare utilization patterns on readmission rates [4]. These findings underscore the importance of comprehensive care models that encompass disease management, preventive strategies, and continuity of care post-discharge to minimize the likelihood of recurrent hospitalizations [5].

Furthermore, the correlation between disease severity and longer hospital stays during readmissions corroborates the notion that advanced CKD stages often entail more complex care needs, resulting in extended hospitalization periods [6]. Addressing the specific needs of patients with advanced CKD, such as enhanced care coordination and specialized interventions, could potentially mitigate the length of hospital stays and improve resource utilization. Interventions aimed at reducing readmissions among CKD patients should be multifaceted, considering the identified predictors and primary causes of readmissions. Transitional care programs focusing on patient education, medication management, and close follow-up post-discharge have shown promise in various chronic disease populations, and their implementation could potentially benefit CKD patients [7]. Additionally, tailored interventions targeting specific high-risk groups, such as elderly CKD patients with multiple comorbidities, might yield substantial reductions in readmission rates [8]. Hospital readmissions among CKD patients pose significant challenges and reflect the intricate interplay of demographic, clinical, and healthcare utilization factors. This research has illuminated critical insights into the determinants and patterns of CKD-related readmissions, providing a foundation for targeted interventions and improved care strategies. The correlation between disease severity, as indicated by advancing CKD stages, and heightened readmission rates underscores the need for proactive management strategies tailored to different disease stages.

Furthermore, the influence of age, comorbid conditions, and prior hospitalizations as predictors of readmissions highlights the importance of personalized care plans addressing individual patient needs. Primary reasons for readmissions, particularly cardiovascular complications and acute kidney injury, emphasize the necessity of comprehensive care models integrating renal and cardiovascular health management [11-15]. Addressing these primary causes through preventive measures and enhanced care coordination could potentially mitigate the burden of recurrent hospitalizations. Transitional care programs, patient education initiatives, and targeted interventions aimed at high-risk groups, such as the elderly with multiple comorbidities, hold promise in reducing readmission rates and improving care outcomes for CKD patients. However, the implementation and evaluation of these interventions in diverse healthcare settings warrant further exploration.

Limitations of this research include its retrospective nature and reliance on data from a single tertiary care unit, which may limit the generalizability of findings to broader CKD populations. Future research encompassing diverse healthcare settings and longitudinal data could provide a more comprehensive understanding of readmission patterns and validate the effectiveness of targeted interventions.

## Conclusions

In conclusion, this research contributes valuable insights into understanding hospital readmissions among CKD patients, emphasizing the need for multifaceted interventions addressing disease severity, comorbidities, and patient-specific factors. Efforts directed at enhancing care continuity, optimizing disease management, and tailoring interventions to individual needs are crucial in reducing readmission rates and improving the quality of care for CKD populations.
